# 我如何治疗人类免疫缺陷病毒感染相关淋巴瘤

**DOI:** 10.3760/cma.j.cn121090-20241108-00441

**Published:** 2025-02

**Authors:** 耀 刘, 超雨 王

**Affiliations:** 重庆大学附属肿瘤医院血液肿瘤中心，肿瘤转移与个体化诊治转化研究重庆市重点实验室，重庆 400030 Hematology-Oncology Center, Chongqing University Cancer Hospital, Chongqing Key Laboratory of Translational Research for Cancer Metastasis and Individualized Treatment, Chongqing 400030, China

## Abstract

人类免疫缺陷病毒（HIV）感染相关淋巴瘤总体发病率低，且可规范收治该类疾病的血液肿瘤专科较少。对于初诊患者，由于获得性免疫缺陷综合征背景下患者免疫功能受损，诱导治疗过程中感染等并发症发生率高，治疗难度增大；对于复发/难治患者，由于缺乏相应的新药临床研究，故挽救治疗方案极其有限。目前国内外临床实践显示，对于HIV感染相关淋巴瘤患者，规范抗逆转录病毒治疗（cART）联合靶向免疫化疗获得完全缓解后尽早行自体造血干细胞移植可使患者获得长期生存。近年来，一些新药（如XPO1抑制剂、免疫检查点抑制剂、抗体药物偶联物、双抗、小分子药物）也尝试加入HIV感染相关淋巴瘤患者的整体治疗方案中并获得了初步的临床数据。本文从本中心收治的2例HIV感染相关淋巴瘤患者出发，提出规范化诊治路径，并在新药应用方面为临床医师提供相应参考，以提升我国HIV感染相关淋巴瘤诊疗水平，提高患者的生存率及生活质量。

## Abstract (en)

The overall incidence of lymphomas associated with HIV infection is low, and only few hematological oncology specialties provide standardized treatment. Newly diagnosed patients with impaired immune function have a high incidence of complications, such as infections during induction chemotherapy, which increases treatment difficulties. For patients with relapse/refractory status, salvage treatment options are extremely limited, because new drug clinical trials are lacking. At present, both domestic and international clinical practices have shown that patients with lymphomas associated with HIV infection can achieve long-term survival after standardized combination antiretroviral therapy（cART）with targeted immunochemotherapy, followed by autologous hematopoietic stem cell transplantation（auto-HSCT）immediately after achieving complete remission. In recent years, some new drugs, such as XPO1 inhibitors, immune checkpoint inhibitors, antibody-drug conjugates（ADC drugs）, bispecific antibodies, and small-molecule drugs, have been tentatively incorporated into treatment regimens for patients with lymphomas associated with HIV infection, and preliminary clinical data have been obtained. Based on two patients with lymphomas associated with HIV infection who were admitted to our center, this study proposed a standardized diagnostic and treatment pathway and provided corresponding references for clinicians to apply new drugs. The aim of this study was to promote diagnostic and treatment capabilities and improve the survival and quality of life of patients of lymphomas associated with HIV infection in China.

随着联合抗逆转录病毒治疗（combination anti-retroviral therapy，cART）的广泛应用，获得性免疫缺陷综合征患者的生存时间得以显著延长[1]。恶性肿瘤已经成为人类免疫缺陷病毒（HIV）感染相关死亡的首要原因。2017年以来，HIV感染相关淋巴瘤的发生率超过卡波西肉瘤，居于HIV感染相关肿瘤的首位，其发病率为（100～300）/100 000（以HIV感染者为基数）[2]。

2022年世界卫生组织（WHO）第五版造血与淋巴组织肿瘤新分类中将HIV感染相关淋巴瘤明确归为独立的一类疾病：与免疫缺陷和失调相关的淋巴组织增生和淋巴瘤。主要亚型包括弥漫大B细胞淋巴瘤（DLBCL）、伯基特淋巴瘤（BL）、原发性中枢神经系统淋巴瘤（PCNSL）、浆母细胞淋巴瘤（PBL）、原发性渗出性淋巴瘤（PEL）和外周T细胞淋巴瘤（PTCL）[3]–[4]。HIV感染相关弥漫大B细胞淋巴瘤（HIV-DLBCL）是HIV感染相关淋巴瘤中最常见的病理亚型，占41％[5]。

获得性免疫缺陷综合征患者免疫功能缺陷使得HIV感染相关淋巴瘤与普通淋巴瘤具有不同的特点，抗淋巴瘤治疗需要密切关注患者免疫缺陷状况。目前对于HIV感染相关淋巴瘤的病因、流行病学、病理生理机制等还缺乏深入的认识，因而在HIV感染相关淋巴瘤的诊断、治疗、预防等策略上仍存在许多挑战。在本文中，我们依据重庆大学附属肿瘤医院的临床工作经验并参考文献资料，对HIV感染相关淋巴瘤的诊治策略进行介绍。

一、典型病例

例1，男，40岁。因“发现腋窝淋巴结肿大20 d”于2022年4月就诊我院。诊断：HIV-DLBCL，Ann Arbor分期为ⅣB期，年龄调整的国际预后指数（aaIPI）评分为3分（高危组）。患者2018年发现感染HIV（男男途径传播），规律服用比克恩丙诺。淋巴瘤确诊时：CD4^+^ T淋巴细胞计数468个/µl，HIV-RNA：未检出（<40拷贝数/ml）。

患者一线予以6个周期R-EPOCH（利妥昔单抗+依托泊苷+泼尼松+长春新碱+环磷酰胺+多柔比星）联合PD1方案化疗，4个周期化疗后PET-CT评估完全代谢缓解（CMR），如图1所示。化疗期间联合鞘内注射4次（阿糖胞苷50 mg+甲氨蝶呤12.5 mg+地塞米松5 mg），脑脊液细胞学及流式细胞术检测均为阴性。患者采集CD34^+^干细胞4.7×10^6^/kg。2022年9月30日予BeEAM（苯达莫司汀+依托泊苷+阿糖胞苷+美法仑）方案预处理，+11 d中性粒细胞植活，+14 d血小板植活。患者靶向化疗及自体造血干细胞移植（auto-HSCT）期间，持续予以复方磺胺甲噁唑预防耶氏肺孢子菌肺炎、阿昔洛韦预防带状疱疹病毒、左氧氟沙星预防细菌感染、氟康唑预防真菌感染。患者目前门诊定期随访，病情持续CR状态，已回归到正常的工作生活当中。

**图1:**
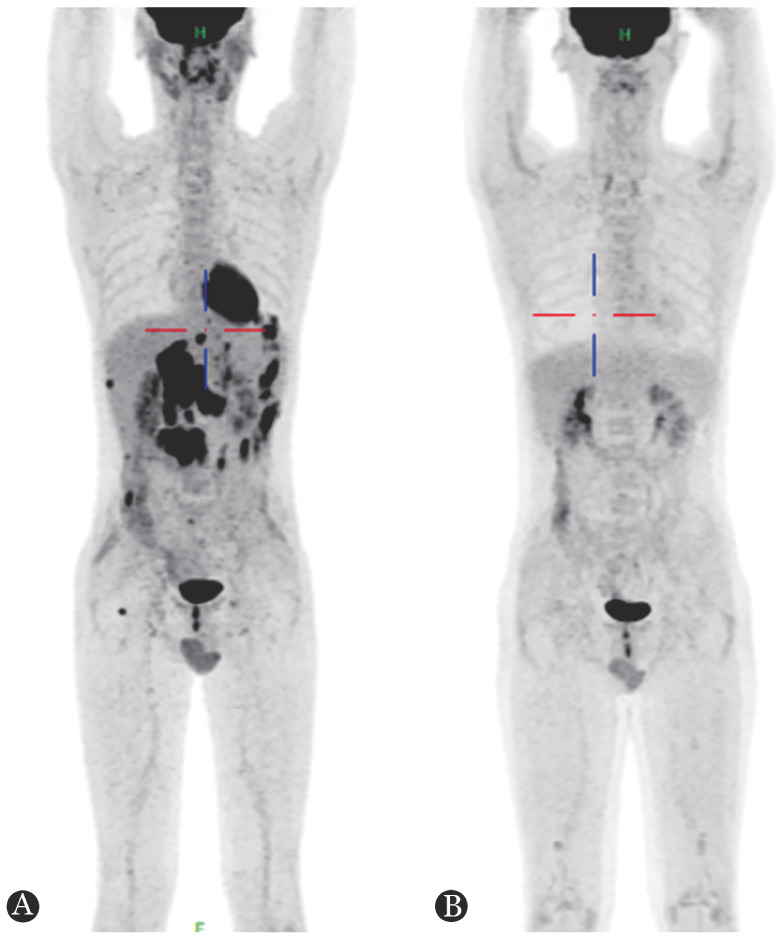
人类免疫缺陷病毒感染相关弥漫大B细胞淋巴瘤患者治疗前后的PET-CT图像 **A** 确诊淋巴瘤时；**B** 4个周期R-EPOCH联合PD1方案化疗后

例2，男，42岁。因“体检发现腹腔肿块4 d”于2024年2月就诊我院。诊断：HIV-BL，Ann Arbor分期为ⅣB期，BL国际预后指数（BL-IPI）：3分（高风险组）。患者在行淋巴结切除活检时查感染标志物发现合并HIV感染（男女途径传播），2024年2月开始规律服用比克恩丙诺。淋巴瘤确诊时：CD4^+^ T淋巴细胞计数52个/µl，HIV-RNA：9.19×10^5^拷贝数/ml。

患者一线予以4个周期R-EPOCH方案化疗，4个周期化疗后PET-CT评估CMR，如图2所示。化疗期间联合鞘内注射4次（阿糖胞苷50 mg+甲氨蝶呤12.5 mg+地塞米松5 mg），脑脊液细胞学及流式细胞术检测均为阴性。2024年5月予以R-EPOCH（第5个周期化疗）动员干细胞，患者采集CD34^+^干细胞7.9×10^6^/kg。2024年5月30日予以第6个周期R-EPOCH方案化疗。2024年7月9日予BeEAM方案预处理，+9 d中性粒细胞植活，+10 d血小板植活。患者靶向化疗及auto-HSCT期间，持续予以复方磺胺甲噁唑预防耶氏肺孢子菌肺炎、阿昔洛韦预防带状疱疹病毒、左氧氟沙星预防细菌感染、氟康唑预防真菌感染。患者2024年10月底复查PET-CT，病情持续CMR状态，CD4^+^ T淋巴细胞计数361个/µl，HIV-RNA：未检出（<40拷贝数/ml）。

**图2:**
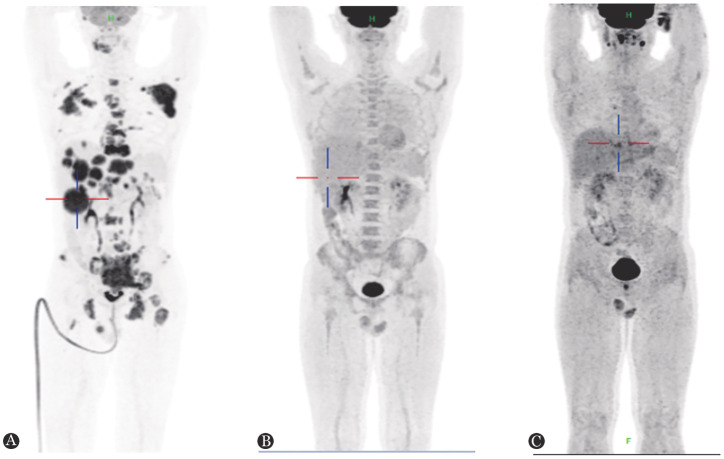
人类免疫缺陷病毒感染相关伯基特淋巴瘤患者治疗前后的PET-CT图像 **A** 确诊淋巴瘤时；**B** 4个周期R-EPOCH方案化疗后；**C** 自体造血干细胞移植3个月后

二、HIV感染相关淋巴瘤的流行病学和临床特点

非霍奇金淋巴瘤（NHL）是获得性免疫缺陷综合征患者死亡的首要原因[6]。20世纪90年代中期引入cART后，HIV感染相关NHL的发病率显著下降，HIV感染相关霍奇金淋巴瘤（HIV-HL）的发病率略有增加[7]–[8]。国外数据显示，HIV感染相关淋巴瘤常见的组织学类型是DLBCL（37％）、HL（26％）和BL（20％）[5],[9]。我国中西部免疫缺陷相关淋巴瘤诊治协作组的数据显示，HIV感染相关淋巴瘤常见的组织学类型分别是DLBCL（67.1％）、BL（11.5％）和HL（4.7％），DLBCL患者的5年总生存（OS）率为54.6％，BL的5年OS率为38.9％[10]。需要注意的是，我们回顾性研究发现56％的HIV感染相关淋巴瘤患者是确诊为淋巴瘤之后发现感染HIV[11]，由此可见，HIV感染相关淋巴瘤的实际发生率缺乏准确的流行病学数据。

HIV感染相关淋巴瘤MYC或BCL6易位及P53突变更常见[12]。临床表现上与普通淋巴瘤患者也存在一定的差异，表现出更高的侵袭性、Ki-67通常>90％、起病年龄更年轻、分期更晚、男性比例明显高于女性[13]–[14]。常伴随B症状、大包块（最大直径≥7.5 cm）及结外受累。最常见的结外受累部位是胃肠道（15％～50％），其他常见累及的结外部位有骨髓（13％～22％）、中枢神经系统（5％～15％）、肝脏（≤5％）和肺（≤5％）[14]。最常见的临床表现为浅表淋巴结肿大，可同时伴有发热、腹痛、骨痛、鼻塞、咳嗽、胸闷、吞咽困难、纳差和乏力等临床表现。

三、HIV感染相关淋巴瘤的发病机制

HIV感染相关淋巴瘤的发生、发展机制并未完全阐明。可能是多因素共同作用的结果，包括：HIV的直接致瘤作用；HIV感染引起的细胞免疫功能缺陷进而导致B淋巴细胞慢性激活；HIV感染背景下致瘤病毒感染风险增加，如EB病毒（EBV）、人类疱疹病毒8型等[15]。重庆大学附属肿瘤医院正在开展相关基础研究（未发表数据），单细胞测序结果显示HIV感染相关淋巴瘤患者更易出现免疫衰老，提示免疫微环境异常可能参与其发病机制。

四、HIV感染相关淋巴瘤的治疗

（一）cART

所有HIV感染相关淋巴瘤患者，在确诊获得性免疫缺陷综合征后都应接受有效的cART，建议终生服用抗逆转录病毒药物，具体用药可参照《中国艾滋病诊疗指南（2024版）》[16]。在化疗期间应继续进行cART，以确保持续的病毒抑制。cART可改善化疗耐受性，有助于免疫功能恢复和患者预后改善。然而，必须考虑免疫化疗药物和抗逆转率病毒药物之间的药代动力学和药效学相互作用。必要时，建议多学科会诊制定cART方案。

（二）感染预防治疗

HIV感染相关淋巴瘤患者一旦出现感染，病情进展迅速，快速获得病原学结果困难，靶向化疗期间的预防性抗微生物治疗十分必要。预防性抗感染策略常需覆盖细菌、耶氏肺孢子菌、带状疱疹病毒。当患者CD4^+^细胞计数<200个/µl时，化疗期间强烈建议使用阿昔洛韦或伐昔洛韦预防带状疱疹病毒，当CD4^+^细胞计数较高时也可以预防使用，因为化疗与泼尼松龙联合使用通常会导致CD4^+^细胞计数下降；所有患者均需要使用复方磺胺甲噁唑预防耶氏肺孢子菌肺炎，当CD4^+^细胞计数<200个细胞/µl时，强烈建议进行预防；建议对接受强化疗的患者进行预防性氟喹诺酮类药物治疗，因为这类患者可能会出现长期（>7 d）中性粒细胞减少症和黏膜炎。通常不建议对真菌进行常规一级预防；然而，对于严重免疫抑制的患者（如CD4^+^细胞计数<100个/µl）、预期长期中性粒细胞减少的患者或接受大剂量甲氨蝶呤（HD-MTX）或阿糖胞苷（HD-Ara-C）方案的患者（最有可能引起黏膜炎），可以考虑使用氟康唑进行抗真菌预防。如果CD4^+^细胞计数<100个/µl，建议动态监测巨细胞病毒变化，必要时给予更昔洛韦、缬更昔洛韦或膦甲酸钠治疗。

对于已明确病原的患者，及时采取针对性的治疗，如针对耶氏肺孢子菌、巨细胞病毒等的治疗。此外，由于多数患者缺乏特异性治疗手段，支持治疗尤其是呼吸支持治疗在重症肺炎的管理中具有重要地位。

（三）抗淋巴瘤治疗

在取得患者充分理解和同意的情况下积极抗淋巴瘤治疗。

1. HIV-DLBCL：如图3所示，对于HIV-DLBCL患者，与HIV阴性DLBCL首选R-CHOP（利妥昔单抗+环磷酰胺+多柔比星+长春新碱+泼尼松）方案不同，其一线治疗方案尚存在争议。本中心前期回顾性研究提示，R-EPOCH方案明显优于R-CHOP方案[13]。近期，中西部免疫缺陷淋巴瘤诊治协作组的回顾性数据提示，4～6个周期的R-EPOCH方案可获得与8个周期R-EPOCH方案同样的生存[17]。本中心开展的一项塞利尼索联合R-EPOCH方案治疗初治HIV-DLBCL的前瞻性、单臂、单中心临床研究，纳入10例患者，客观缓解率（ORR）为100％、CR率为80％，初步体现了新药的加入可进一步改善患者疗效。因此，在无活动性感染的前提下，建议一线给予4～6个周期R-EPOCH方案化疗，期间行4次鞘内注射，后续行auto-HSCT巩固治疗。目前对于HIV-DLBCL维持治疗数据较少，一般不推荐维持治疗。本中心正在开发一种针对HIV-DLBCL患者的预后模型（未发表）——ARDPI（https://cqardlbcl.shinyapps.io/cqardlbcl/），主要用于新诊断HIV-DLBCL患者的预后评估及治疗指导等，具体包括以下7个因素：年龄、淋巴细胞/单核细胞比率（LMR）、淋巴瘤细胞CD5表达、血液EBV-DNA拷贝数、CD4/CD8比率、中枢神经系统受累和获得性免疫缺陷综合征抗逆转录病毒治疗。根据以上7个因素可将新诊断HIV-DLBCL患者分为低危、中危及高危，其预测精度优于IPI和NCCN-IPI，且具有更高的临床获益。根据ARDPI预后分层对患者实施个体化精准治疗，如对高危患者采用治疗强度更大的方案等，有望改善HIV-DLBCL患者的整体预后。

**图3:**
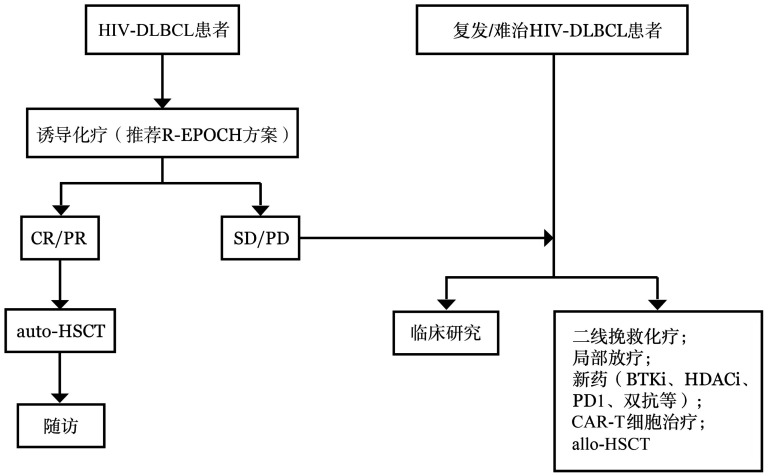
人类免疫缺陷病毒感染相关弥漫大B细胞淋巴瘤治疗路径 **注** HIV-DLBCL：人类免疫缺陷病毒感染相关弥漫大B细胞淋巴瘤；R-EPOCH：利妥昔单抗+依托泊苷+泼尼松+长春新碱+环磷酰胺+多柔比星；CR：完全缓解；PR：部分缓解；SD：疾病稳定；PD：疾病进展；BTKi：布鲁顿酪氨酸激酶抑制剂；HDACi：组蛋白去乙酰化酶抑制剂；PD1：程序性死亡受体1；CAR-T细胞：嵌合抗原受体T细胞；auto-HSCT：自体造血干细胞移植；allo-HSCT：异基因造血干细胞移植

2. HIV-BL：对于HIV-BL患者，我中心前期回顾性分析17例HIV-BL患者，1年OS率仅47.6％[18]。由于侵袭性强，更易合并中枢神经系统受累，建议所有患者基线做腰椎穿刺，脑脊液进行流式细胞术检测，同时使用甲氨蝶呤鞘内注射，脑脊液阳性患者应每周鞘内注射1次甲氨蝶呤，直至脑脊液流式细胞术检测阴性；HIV-BL应采用与HIV阴性患者相同的多药方案进行治疗，多数采用DA-EPOCH-R（剂量调整的依托泊苷+泼尼松+长春新碱+环磷酰胺+多柔比星+利妥昔单抗）、R-CODOX-M/R-IVAC（利妥昔单抗+环磷酰胺+长春新碱+多柔比星+甲氨蝶呤）/（利妥昔单抗+异环磷酰胺+依托泊苷+阿糖胞苷）方案化疗。

3. HIV-HL：患者总体预后良好，我中心回顾性分析一线采用ABVD（多柔比星+博来霉素+长春新碱+达卡巴嗪）方案化疗的22例患者，随访46.8个月，5年无进展生存率为83.9％，5年OS率为89.5％[19]。局限期HIV-HL患者应接受2～4个周期的ABVD方案治疗；中晚期HIV-HL患者可以接受4～6个周期的ABVD方案治疗，然后对PET-CT阳性病灶（残留淋巴结长径≥2.5 cm）进行放疗；新药CD30单抗的联合治疗（BV-AVD）可作为新诊断的Ⅳ期患者的一线治疗方案选择。

4. HIV-PCNSL：其发生在严重免疫功能低下的患者中，治疗原则同HIV阴性的PCNSL一致，推荐使用利妥昔单抗+大剂量甲氨蝶呤（≥3 g/m^2^）化疗；对于HIV控制良好的患者，应考虑多药诱导方案［如MATRix（甲氨蝶呤+阿糖胞苷+塞替哌+利妥昔单抗）］和auto-HSCT巩固；对于化疗不敏感或不适合免疫化疗的患者可考虑使用全脑放疗。

5. auto-HSCT：国外多项临床研究结果显示，auto-HSCT可作为复发/难治（R/R）HIV感染相关淋巴瘤安全、有效的挽救治疗手段[20]–[21]。国内包括北京肿瘤医院、云南霍普禾森医院、甘肃武威人民医院、南方医科大学附属南方医院、西南医科大学附属医院等中心均开展过HIV感染相关淋巴瘤的auto-HSCT[22]。本中心前期共完成HIV感染相关淋巴瘤患者auto-HSCT 19例次，其中2例为双次移植。疾病类型包括DLBCL 8例、BL 6例、PBL 3例、高级别B细胞淋巴瘤（HGBL）1例、R/R HL 1例；中位采集CD34^+^干细胞4.7（2.2～12.19）×10^6^/kg。其中14例患者为一线auto-HSCT巩固，中性粒细胞植入中位时间为11（9～22）d，血小板植入中位时间为15（10～41）d。无移植相关死亡，2例auto-HSCT前未达CR患者因疾病进展死亡。随访数据显示，自2021年首例HIV感染相关淋巴瘤患者完成auto-HSCT以来，所有一线行auto-HSCT巩固的患者均良好生存（数据待发表）。因此，我们推荐临床实践中对HIV感染相关侵袭性淋巴瘤采用强化疗联合auto-HSCT巩固的综合策略治疗。

6. 新药探索：目前关于HIV感染相关淋巴瘤的临床研究尚少，其中基因治疗、BTK抑制剂（BTKi）、组蛋白去乙酰化酶抑制剂（HDACi）、嵌合抗原受体T细胞（CAR-T细胞）治疗等正在临床研究阶段。本中心开展的一项塞利尼索联合R-EPOCH方案治疗初治HIV-DLBCL的临床研究显示ORR为100％、CR率为80％，且安全可控，初步体现了新药的加入可进一步提高患者疗效[23]。目前本中心正在开展CD20/CD3双抗在R/R HIV感染相关B细胞淋巴瘤中的临床研究，探索新药在R/R患者中的疗效。另外免疫调节剂联合PD1单抗、坦昔妥单抗（Tafasitamab）、Loncastuximab、BCL2抑制剂等亦可探索。强烈呼吁未来的临床研究不应仅因患者合并HIV感染而排除其入组，对于HIV-RNA转阴，CD4^+^细胞计数>200个/µl的非活动期HIV感染者应纳入临床研究。

五、展望及未来研究方向

近年来，HIV感染相关淋巴瘤规范化诊疗在我国专业领域学者的努力下已有长足进步，如规范cART联合免疫靶向治疗，另外，一些新的治疗方案如新药（XPO1抑制剂、PD1抑制剂、CD30单抗等）的加入、auto-HSCT也取得了相应临床疗效。然而还有很多基础及临床问题尚待解决：①如何使HIV感染相关淋巴瘤患者也能受益于新药临床试验，不能仅因合并HIV感染而排除经过规范的抗病毒治疗病毒转阴、免疫功能重建的患者；②深入研究HIV感染相关淋巴瘤的病理生理机制，探寻新的治疗靶点；③探讨HIV感染相关淋巴瘤的预防策略（病毒疫苗或促进免疫重建等）。

总之，HIV感染相关淋巴瘤的流行病学、快速诊断手段、预后评估指标、特异性治疗策略等方面依然还需要更多更深入的研究。而上述问题的研究将有助于进一步理解HIV感染相关淋巴瘤的病理生理过程并有助于优化HIV感染相关淋巴瘤的防治策略。
